# Long gun violence in California versus Texas: How legislation can reduce firearm violence

**DOI:** 10.1016/j.sopen.2024.05.011

**Published:** 2024-05-29

**Authors:** Jonathan Shipley, Areg Grigorian, Lourdes Swentek, Cristobal Barrios, Catherine Kuza, Jeffrey Santos, Jeffry Nahmias

**Affiliations:** a University of California, Irvine, Department of Surgery, Division of Trauma, Burns and Surgical Critical Care, Orange, CA, USA; b Keck School of Medicine of the University of Southern California, Department of Anesthesia, Los Angeles, CA, USA

## Abstract

**Introduction:**

Long guns (LGs) are uniquely implicated in firearm violence and mass shootings. On 1/1/2019 California (CA) raised the minimum age to purchase LGs from 18 to 21. This study aimed to evaluate the incidence of LG violence in CA vs. Texas (TX), a state with rising firearm usage and fewer LG regulations, hypothesizing decreased LG firearm incidents in CA vs increased rates in TX after CA LG legislation.

**Methods:**

A retrospective analysis of the Gun Violence Archive (2015–2021) was performed. An additional analysis of all firearm incidents within TX and CA was performed. CA and TX census data were used to calculate incidents of LG violence per 10,000,000 people. The primary outcome was the number of LG-related firearm incidents. Median yearly rates of LG violence per 10,000,000 people were compared for pre (2015–2018) vs post (2019–2021) CA LG legislation (Senate Bill 1100 (SB1100).

**Results:**

Median LG incidents decreased in CA post-SB1100 (4.21 vs 1.52, *p* < 0.001) by nearly 64 %, whereas any gun firearm violence was similar pre vs post-SB1100 (77.0 vs 74.5 median incidents, *p* = 0.89). In contrast, median LG incidents increased after SB1100 (4.34 vs 5.17 median incidents, *p* = 0.011) by nearly 35 % in TX, with any gun incidents increasing by nearly 53 % (83.48 vs 127.46, p < 0.001).

**Conclusion:**

CA LG firearm incidents decreased following SB 1100 legislation whereas the incidence in TX increased during this same time. Meanwhile, the incidence of any firearm violence remained similar in CA but increased in TX. This suggests the sharp decline in CA LG incidents may be related to SB1100. Accordingly, increasing the age to purchase a LG from 18 to 21 at a federal level may help curtail LG violence nationally.

## Introduction

The United States (US) possesses the greatest number of privately owned firearms in the world, with more firearms than citizens within its borders [1]. This immense number of firearms is not without consequence, as firearm violence is responsible for over 67,000 injuries and >32,000 deaths every year [2]. A significant proportion of these deaths are suicides, as the US contributes to over 35 % of global firearm suicides despite only accounting for ∼4 % of the world's population [3]. Moreover, with the expiration of the federal assault rifle ban in 2004, the arsenal of weapons owned in the US is growing in lethality, and since 2004 there has been a surge in assault rifle firearm violence [4]. In fact, it has been reported there would be 70 % fewer mass shooting fatalities if the assault rifle ban were still in place [5]. The increasing number of deaths and injuries related to firearm violence has placed an immense burden on the US healthcare system, with some estimates indicating that the cost of treating firearm-related injuries is nearly $2.3 billion every year [1]. Given the frequency and volume of firearm-related mortality and injury, firearm violence has become a public health crisis [2,6,7].

Unfortunately, discussing firearm legislation can evoke contentious political debate centered around America's Second Amendment right [1,8]. Because of the political nature of firearm violence in the US, federal research that may help inform legislation and federal policies has been significantly hindered [8]. A 2018 study found that the Centers for Disease Control and Prevention (CDC) allocated <0.1 % of its annual budget to firearm violence research [8]. However, it has been previously demonstrated that stronger firearm policies and legislation can reduce firearm incidents, injuries, homicides, and suicides [[9], [10], [11], [12]].

One specific type of firearm that has garnered increased attention includes long guns (LGs). It is estimated that 25 % of mass shooting homicides and over 75 % of mass shooting injuries occur secondary to LGs [5]. Despite this, LGs are significantly less regulated when compared to handguns, even though LGs represent up to 45 % of firearm suicides in rural states and in younger populations [13]. Such statistics are of particular concern as the national minimum age for purchasing a LG from a licensed dealer is just 18 years old, while the minimum age for purchasing a handgun is 21 [14]. Moreover, recent mass shootings such as those in Uvalde, Texas, Parkland, Florida, and Buffalo, New York, all involved a LG that the perpetrator bought legally before turning 21 [15]. In 2018, the state of California (CA) responded by implementing legislation that raised the minimum age to purchase a LG from 18 to 21 years old. This law, known as Senate Bill 1100 (SB1100), went into effect on January 1st, 2019, and it also limited the maximum number of LG purchases to just one per month [16]. As it has been demonstrated that raising the minimum age to purchase a handgun from 18 to 21 leads to a decrease in suicides by handgun, increasing the age to purchase a LG may have similar impacts on young adult firearm violence, including suicides [14]. Therefore, this study sought to evaluate the incidence of LG violence before and after the implementation of SB1100 in CA compared to Texas (TX), a state with a persistent rise in firearm usage and minimal LG regulations, hypothesizing increased rates of LG firearm incidents in TX and decreased rates in CA after the implementation of SB1100 in 2019.

## Methods

This study was deemed exempt by the institutional review board and a waiver of consent was granted due to the use of a de-identified national database. A retrospective analysis of the Gun Violence Archive (GVA), an independent and online organization that uses automated internet inquiries to gather incidents of firearm violence across the US from >7500 sources, was performed for the years 2015–2021 [17]. This database is free to use and accessible to the public. Incidents involving any LG (defined as a firearm with a long barrel such as a rifle or shotgun) were included. Incidents were defined as any firearm violence in which a LG was used, including fatal and non-fatal incidents. An additional analysis of all firearm incidents within TX and CA was performed to provide a perspective of non-LG firearm trends. To control for changes in population across various years, CA and TX census data were used to calculate incidents of LG violence per 10,000,000 people. The primary outcome was the number of LG-related firearm incidents. Mann-Whitney *U* tests were used to compare median yearly rates of LG violence per 10,000,000 people before (2015, 2016, 2017, and 2018) versus after (2019, 2020, and 2021) SB1100 went into effect. A *p*-value <0.05 was considered statistically significant. All analyses were performed using IBM SPSS Statistics, Version 26 (IBM Corp., Armonk, New York).

## Results

**Fig. 1:**
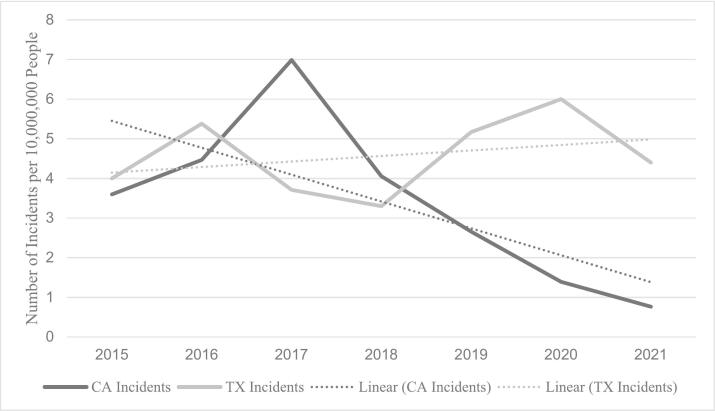
Median yearly incidence of long gun incidents in California and Texas per 10,000,000 people between 2015 and 2021.

In CA, there were 160 LG shootings in 2015, 205 in 2016, 312 in 2017, 230 in 2018, 116 in 2019, 71 in 2020, and 41 in 2021. Median LG incidents per 10,000,000 people decreased significantly after SB1100 (4.21 vs 1.52 median incidents, *p* < 0.001) (Table 1). This represents a 63.9 % decrease in LG violence in CA following the implementation of SB1100. However, firearm violence due to any gun in CA did not change when comparing before versus after SB1100 (77.04 vs 74.53 median incidents, *p* = 0.892) (Fig. 1).

**Table 1:** Mann-Whitney *U* Test for Median Year Rates of Long Gun and Any Gun Incidents per 10,000,000 People (Before vs. After 2018) in California and Texas.

	Incidents before law	Incidents After law	p
Long gun incidents			
California (IQR)	4.21 (3.83)	1.52 (1.24)	**<0.001**
Texas (IQR)	4.34 (3.64)	5.17 (3.74)	**0.011**
Any gun incidents			
California (IQR)	77.04 (20.3)	73.53 (18.4)	0.892
Texas (IQR)	83.48 (14.7)	127.5 (37.3)	**<0.001**

**Fig. 2:**
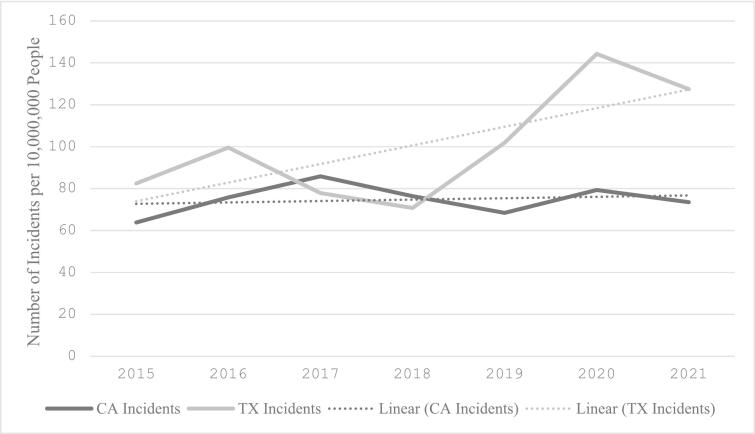
Median yearly incidence of any gun incidents in California and Texas per 10,000,000 people between 2015 and 2021.

In contrast, in TX there were 136 LG shootings in 2015, 189 in 2016, 152 in 2017, 118 in 2018, 192 in 2019, 198 in 2020, and 164 in 2021. Median LG incidents per 10,000,000 increased from pre to post-SB1100 (4.34 vs 5.17 median incidents, *p* = 0.011). This demonstrates a 34.5 % increase in LG violence. In terms of any gun violence in TX, incidents also increased during this time period (83.48 vs 127.46 median incidents, *p* < 0.001) (Fig. 2).

## Discussion

Firearm violence is the leading cause of death for Americans under 24 [18]. In the past two decades, the rate of mortality due to firearms in CA has decreased by >50 % which is almost 4× the decrease seen in the rest of the country [19]. Many of CA's most important firearm laws went into effect during this time [1,2,19]. This study adds to this trend as we found that LG violence decreased in CA after SB1100, despite CA experiencing no overall change in any gun firearm violence during this same time period. In contrast, TX saw a significant increase in both LG incidents and any gun incidents. Accordingly, the decrease in LG incidents in CA may be attributable to the implementation of SB1100 legislation.

Decreased LG violence in CA after SB1100 might have been due to a decrease in LG suicides among young adults, as this population could no longer acquire a LG to undergo suicide by firearm after the law went into effect [20]. As previously mentioned, states that have increased the age to purchase a handgun from 18 to 21 experienced a decrease in both young adult and adolescent suicides, and this may also be true in the case of LGs [14]. Historically, youth suicides represent a substantial proportion of firearm deaths, estimated at over 40% [21]. Young adult populations are more likely to use a LG in firearm suicide when compared to older adults, which may be due to LGs being more accessible than handguns that have a federal age limit of 21 [13]. In fact, one study found that the number of youth suicides by LG was nearly 45 % whereas older adults (65 and older) used LGs for suicide only about 20 % of the time [13]. This may be due to the federal age limit needed to purchase a LG being 18, as well as the fact that LGs are often less expensive than handguns [13,22]. Further contributing to LG accessibility is the fact that there is no national minimum age requirement for purchasing a LG from an unlicensed dealer [3,23]. This has prompted some states to allow unlicensed dealers to legally sell LGs to individuals as young as 16, potentially further contributing to the use of LG suicide in younger populations [3,23].

SB1100 may have also reduced LG violence by reducing the number of LGs owned by adults under 21, a population that has been shown to participate in riskier firearm activities [5,16]. Such activities include carrying a weapon while intoxicated, firearm aggression, and firearm discharge in high-risk situations such as committing a crime [5]. Also, while only a small proportion of firearm violence results from mass shootings, laws like SB1100 may prevent LGs from being purchased legally to commit mass murder [24]. One study found that 25 % of school shootings involving LGs over the last 36 years were committed by individuals under 21 who bought their firearms legally [24]. Thus, this study has potential public health implications for non-firearm users, as it reveals how policy may directly impact firearm violence. Specifically, this study provides further evidence that raising the age to purchase a LG from 18 to 21 at a federal level may reduce firearm violence.

One of the most obvious yet crucial factors contributing to firearm violence is access to firearms [19]. While we report a significant decrease in LG violence in CA after SB1100, we also found that there was a large increase in LG violence in CA between 2015 and 2017, leading up to SB1100. Data within the CA Department of Justice demonstrates the number of firearms, including LGs, purchased in 2016 was significantly higher than the years prior, or after, which may contribute to the year of most incidents in 2017 [25].

Our study is inherently limited by factors associated with its retrospective design and use of the GVA database. This includes both misclassification errors and unreported incidents. The GVA works by reporting automated internet inquiries. Regions of the US in which there remains less access to police departments or other resources capable of reporting incidents of LG violence are likely to suffer from underreporting of incidents. Further, as incidents were found using weapon type as a search rule, incidents categorized incorrectly by weapon type, or those in which incidents did not specify the weapon used in incidents, may have led to unreported incidents of LG violence. The number of firearm incidents reported in the GVA every year varies, with the total number of all-cause firearm violence-related deaths estimated between 40,000 and 50,000 per year [17]. Though the GVA has been validified as an epidemiologic study tool, it does underreport certain crimes such as those involving African Americans who are injured. Despite this, the estimated sensitivity of the GVA was between approximately 70 and 90 %, with increases in sensitivity over time. The positive predictive value has also been shown to be as high as 99 % [17,26]. It is worth mentioning that CA implemented additional LG-related laws around this study's time period, including a law that went into effect in 2015, mandating any LG purchaser to obtain a firearm safety certificate to prove their awareness of firearm safety [25]. If the US increased the minimum age to purchase a LG from 18 to 21 federally, it is important to consider that such legislation may lead to aggressive behavior that manifests through alternative means in response to limitations on firearm availability. More specifically, a displacement effect may develop in which individuals who are no longer able to obtain firearms resort to other methods of violence. However, future research is necessary to ascertain the dynamics between firearm legislation and overall violence trends. Finally, while our study provides evidence regarding trends in firearm violence in TX and CA, our analysis does not encompass a comparative examination of firearm violence across the remaining US states. Accordingly, we cannot conclusively determine whether the increased violence experienced in TX can be further extrapolated to the remaining states and the US as a whole, and thus attributing changes in firearm violence rates solely to CA legislation may oversimplify complex factors that influence firearm violence in each state. Caution should be exercised when drawing direct conclusions regarding the efficacy of legislation on overall firearm violence rates, and future research is required to explore these trends more comprehensively.

## Conclusion

This retrospective analysis of LG violence in CA and TX spanning seven years of data found a significant decrease in LG violence in CA after the implementation of SB1100, despite there being no change in any gun firearm violence in CA. During this same time, TX had an increase in both LG violence and firearm violence from any firearm. While this decrease in CA is likely attributable to SB1100, future research is needed to confirm this finding. This would best be accomplished by states like TX trialing similar legislation to determine if it is generalizable and would be beneficial as a federal legislative change.

## Funding sources

NA.

## Ethics approval

This project had no work on human beings. NA.

## CRediT authorship contribution statement

**Jonathan Shipley:** Writing – review & editing, Writing – original draft, Investigation, Data curation, Conceptualization. **Areg Grigorian:** Writing – review & editing, Writing – original draft. **Lourdes Swentek:** Writing – review & editing, Visualization, Supervision. **Cristobal Barrios:** Writing – review & editing, Supervision, Methodology. **Catherine Kuza:** Writing – review & editing. **Jeffry Nahmias:** Writing – review & editing, Writing – original draft, Validation, Supervision, Investigation, Conceptualization.

## Declaration of competing interest

The authors have no financial or personal relationships with other people or organizations that could inappropriately influence their work. NA.
